# Comparison of Band Percentage vs Immature Granulocyte Percentage in the Setting of Possible Infection Principle Investigators

**DOI:** 10.1155/ah/3616532

**Published:** 2024-12-30

**Authors:** Daniel Hershberger, Kaeli Samson, M. Ahsan Saeed

**Affiliations:** ^1^Department of Internal Medicine, Division of Pulmonary and Critical Care, University of Nebraska Medical Center, Omaha, Nebraska, USA; ^2^Department of Biostatistics, College of Public Health, University of Nebraska Medical Center, Omaha, Nebraska, USA; ^3^Department of Pulmonary and Critical Care, Elkhart General Hospital, Elkhart, Indiana, USA

## Abstract

**Background:** Sepsis is a major cause of mortality worldwide. Early identification and treatment are critical to improve survival. Band count has been used as part of SIRS criteria for the early identification of potentially septic patients. Unfortunately, band count requires manual interpretation. This leads to increased potential for intraobserver variability. Immature granulocytes are counted in an automated fashion, which has the potential to improve accuracy and reduce costs.

**Research Objective:** We aim to compare the band percentage and immature granulocyte percentage to set the threshold for immature granulocyte percentage equal to the sensitivity of > 10% band count.

**Methods:** A retrospective chart review was conducted at a single academic medical center. Data from patients with SIRS criteria and measured immature granulocyte and band percentages were utilized to explore potential associations between immature granulocyte percentages and band percentages. Data were analyzed using Spearman correlations, Wilcoxon rank-sum tests, and logistic regressions.

**Results:** We found no significant associations between immature granulocyte percentage and band percentage or other SIRS criteria.

**Conclusion:** We conclude that immature granulocyte percentage does not correlate with band percentage in the setting of possible infection.

## 1. Background

The American College of Chest Physicians/Society of Critical Care Medicine's systemic inflammatory response syndrome (SIRS) criteria have been used in clinical medicine since their consensus statement was released in 1992 [1]. SIRS criteria are widely accepted as overly sensitive and nonspecific. SIRS criteria contain four parts: body temperature > 38  C or < 36  C; heart rate > 90 beats per minute; tachypnea with a respiratory rate > 20 breaths per minute or hyperventilation with a PaCO_2_ < 32 mmHg; and white blood cell count > 12,000 cu mm or < 4000 cu mm or > 10% immature neutrophils—also known as bands. To meet SIRS criteria, a patient must have 2 of 4 parts.

Band count as an individual test has a sensitivity of 43% and a specificity of 92% [2]. It has also been used in a study to predict outcomes in severe acute pancreatitis [3]. Band counts require a manual differential with a cytotechnologist counting the number of bands seen. Intraobserver variability likely occurs but has not been well studied. Currently, no automated process exists to evaluate for bands.

Immature granulocyte counts are comprised of promyelocytes, myelocytes, and metamyelocytes, which are the immediate precursors of bands. Several laboratories are utilizing automated immature granulocyte counts instead of manual band counts. This transition is based on several articles that propose that immature granulocyte count and percentage can be used as biomarkers for sepsis. Unfortunately, immature granulocyte count suffers from an overall poor sensitivity when the reference value is set too high and poor specificity when the reference value is set too low [4–7].

There is a paucity of studies evaluating the level of immature granulocyte percentage that is equivalent to > 10% band count. As one of the main components of a complete blood count with differential, band count and immature granulocyte count are used daily by clinicians to assess for infection. The further understanding of these parameters and how they correlate is valuable.

## 2. Methods

Study Population: This study was conducted at the University of Nebraska Medical Center in Omaha, Nebraska, USA. IRB approval was granted for this retrospective review. Inclusion criteria included all patients aged 19 years and older presenting with two or more SIRS criteria and concurrent measurements of bands and immature granulocytes. Automated hematologic studies were analyzed on the Sysmex XN9000. Certified medical laboratory scientists performed manual 100-cell differentials with band count.

## 3. Study Design

All instances of patients aged 19 years and older who presented to the emergency department between January 1, 2014, and January 1, 2018, and were admitted or transferred from outside facilities, met the two elements of SIRS criteria, and had measures for both band count and immature neutrophil count were selected for this study. There were 4636 patients with SIRS criteria identified during this time frame. Patients whose first SIRS instance occurred within the first month of the study period were excluded (*n* = 81) in the event they had received antibiotics before the data collection period. We selected patients who had their band count measured within the 48 h prior to having their first SIRS criteria met (*n* = 2410). Lastly, we selected patients who had immature granulocyte count values available within ± 24 h of the band count measurement. The final sample size was 1369 patients.

The raw value data were analyzed. Due to the skewed, nonnormal distribution of the data, statistics were also analyzed after log-transformations. Log-transformation was performed after adding a value of one to the raw data since some variables had values of zero. To assess the association between band percentage and immature granulocyte percentage, Spearman correlations were calculated or Pearson correlations were calculated if data were log-transformed. To calculate an area under the receiver operator characteristic (ROC) curve estimate, raw band percentage was dichotomized at 10% (i.e., ≤ 10 vs. > 10) and that variable was used as the outcome in a logistic regression where log immature granulocyte percentage was the only predictor.

For exploratory analyses, we looked at patterns of the association between band percentage or immature neutrophil percentage and 30-day mortality (defined from the first date the SIRS criteria were met). Jitter was used on scatter plots to help visualize overlapping values. A Wilcoxon rank-sum test was used to assess the difference in immature granulocyte percentage between patients who did or did not survive 30 days after the initial SIRS criteria. Logistic regression was also used to determine associations between log immature granulocyte percentage and 30-day mortality. All analyses were performed using SAS software Version 9.4 (SAS Institute Inc., Cary, NC).

## 4. Results

Of 1369 patients included in the analysis, 763 were male (55.7%). The average age was 55.9 years. Thirty-day mortality was observed in 163 patients (11.9%).

**Figure 1:**
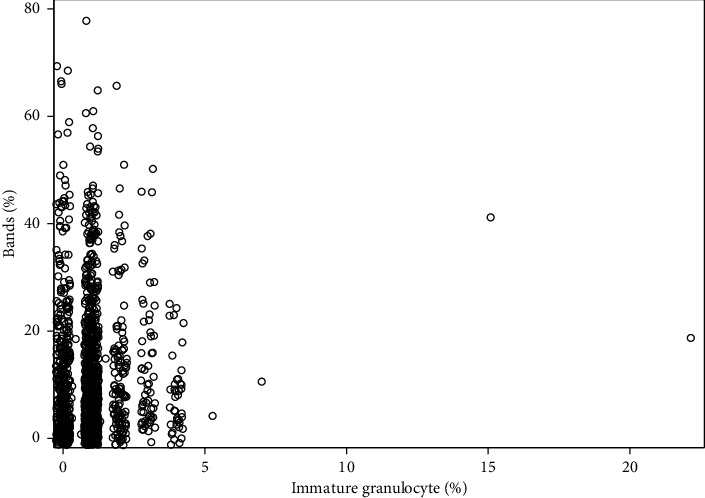
Scatter plot of raw immature granulocyte (%) by raw bands (%).

When evaluating the raw values, there was no statistically significant correlation between band percentage and immature granulocyte percentage (Spearman *ρ* = 0.04, *p*=0.14) (Figure 1). When the raw values were log-transformed, the correlation only slightly increased (Pearson *r* = 0.05; *p*=0.05).

**Figure 2:**
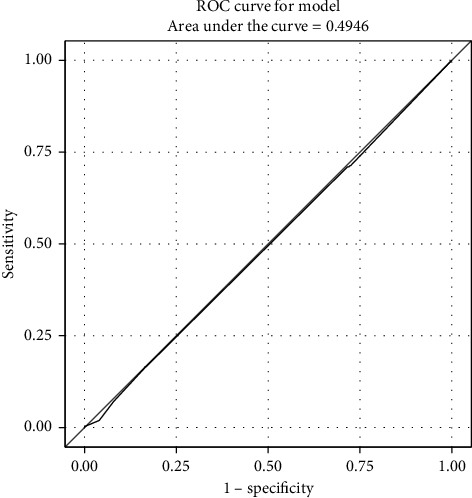
A receiver operator characteristic curve for a logistic model where dichotomous band percentage was the outcome (greater than 10%, or less than or equal to 10%) and raw immature granulocyte was the only variable in the model. A value of 0.50 is equivalent to chance, while a value of 1.00 represents perfect classification.

To assess the performance of immature granulocyte percentage to predict > 10% band percentage, band percentage was dichotomized into two groups: greater than 10%, or less than or equal to 10%. Logistic regression, with the dichotomous band percentage greater than 10% as the outcome and raw immature granulocyte percentage as the only variable in the model, generated an area under the ROC curve of 0.49, indicating poor performance of raw immature granulocytes to classify > 10% bands (Figure 2). A similar model was run to assess the log-transformed immature granulocyte percentage, and this model also demonstrated poor statistical performance (area under the ROC curve = 0.51).

**Figure 3:**
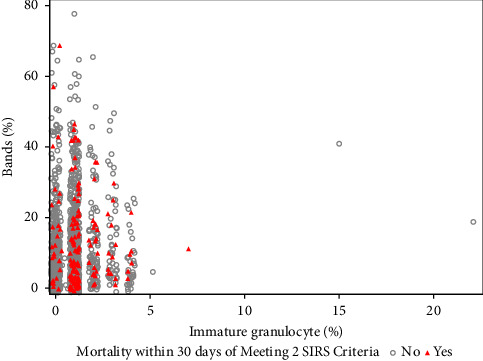
Scatter plot of immature granulocyte (%) by band (%). Patients who died within 30 days of first meeting SIRS criteria are indicated in red triangles, while those who did not die within 30 days are indicated with circles.

**Figure 4:**
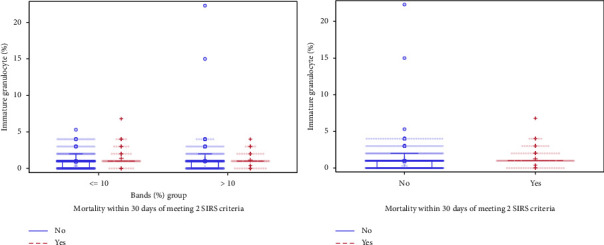
Vertical box plots of immature granulocyte (%) by 30-day mortality (where the 30-day period starts when patients first meet SIRS criteria).

The association between immature granulocyte percentage and band percentage does not appear to differ based on 30-day mortality, as seen in Figure 3. However, regardless of dichotomous band grouping, patients who died within 30 days from the time of 2 SIRS criteria being met had elevated immature granulocytes relative to those who survived (*p* < 0.001; Figure 4).

## 5. Discussion

While band count has been shown to provide clinically relevant data in prior studies concerning sepsis, several factors make it less dependable. Band counts are done manually and may suffer from interobserver variability. As laboratories and clinical practices weigh the possibility of transitioning from manual band counts to automated immature granulocyte counts, the differences between the two values need to be considered. If a transition to automation is performed correctly, the result could potentially decrease costs and improve reliability.

No prior studies have explored the possibility of using the automated immature granulocyte percentage as a substitute for the band percentage [8]. Our study is unique in evaluating this relationship. Our data suggest that despite immature granulocytes being the precursor to bands, the percentage of immature granulocytes does not correlate with the percentage of bands observed.

Recent research suggests that immature granulocytes may help differentiate between septic and nonseptic patients with high sensitivity and specificity [9]. Immature granulocyte count has also been analyzed as a potential biomarker for bacteremia [10]. Elevated myelocytes and metamyelocytes could potentially be utilized for prognostic purposes [11]. Our data suggest that immature granulocyte percentage may serve as a marker for risk of mortality, but further multicenter research needs to be conducted to evaluate this further.

Adjusting laboratory cutoffs for immature granulocyte percentage and count results in different predictive values and limits ease of use [12]. Some studies have validated a cutoff of 3.2% for immature granulocytes as optimal for diagnosing sepsis [13]. The ideal value remains to be determined.

The limitations of our study include the fact that it is a single-center study. This was a retrospective analysis, and despite bands and immature granulocytes being measured within 24 h of each other, the laboratory collection was not necessarily concurrent. Despite this, a dedicated effort was made to limit the confounding factors, including excluding patients who met SIRS criteria one month before the study as they had potentially received antibiotics. The dataset is also unique as it comes before the COVID-19 pandemic and represents patients mostly with sepsis due to other causes than COVID-19 pneumonia. Data samples during the pandemic may have to account for the leukopenia seen in SARS-CoV-2 infections.

## 6. Conclusion

Immature granulocyte percentage does not appear to correlate with band percentage. Additional research is needed into immature granulocyte percentage as it relates to sepsis recognition.

## Data Availability

The data that support the findings of this study may be made available from the corresponding author upon reasonable request.
